# Analysis of Hepatitis B Virus Intrahepatic Covalently Closed Circular DNA and Serum Viral Markers in Treatment-Naive Patients with Acute and Chronic HBV Infection

**DOI:** 10.1371/journal.pone.0089046

**Published:** 2014-02-13

**Authors:** Weijie Li, Jingmin Zhao, Zhengsheng Zou, Yan Liu, Baosen Li, Ying Sun, Xiaodong Li, Shuhong Liu, Shaoping Cai, Weimin Yao, Shaojie Xin, Fengmin Lu, Dongping Xu

**Affiliations:** 1 Department of Microbiology, Peking University Health Science Center, Beijing, China; 2 Institute of Infectious Diseases and Medical Center for Liver Failure, Beijing 302 Hospital, Beijing, China; 3 Department of Pathology, Beijing 302 Hospital, Beijing, China; Drexel University College of Medicine, United States of America

## Abstract

**Background:**

This study aimed to investigate the relationships of intrahepatic cccDNA with serum HBsAg and with HBV DNA in treatment-naive patients throughout acute and chronic HBV infection.

**Methods:**

A total of 120 patients who had a liver biopsy were enrolled, including 19 with acute hepatitis B (AHB), and 101 patients with chronic HBV infection (CHB) of whom were 10 in immune-tolerant (IT) phase, 59 in immune-clearance (IC) phase, 8 in low-replicative (LR) phase, and 24 in HBeAg-negative hepatitis (ENH) phase. Intrahepatic cccDNA, serum HBsAg and serum HBV DNA levels were comparatively analyzed.

**Results:**

The median intrahepatic cccDNA levels were 0.18 4.80, 3.81, 0.22 and 0.97 copies/cell for patients with AHB, CHB-IT, CHB-IC, CHB-LR, and CHB-ENH, respectively. In AHB patients, intrahepatic cccDNA was positively correlated with serum HBsAg (*r* = 0.665, *P* = 0.003), as well as serum HBV DNA (*r* = 0.536, *P = *0.022). In CHB patients, intrahepatic cccDNA was positively correlated with serum HBsAg in the IC phase (*r* = 0.392, *P = *0.005), and with serum HBV DNA in the IC phase (*r* = 0.301, *P = *0.036) and ENH phase (*r* = 0.588, *P = *0.013). HBV replicative efficiency, defined as the ratio of serum HBV DNA to intrahepatic cccDNA, was obviously lower in AHB and CHB-LR patients than in CHB-IT, CHB-IC and CHB-ENH patients (0.70 and 0.53 vs. 1.12, 1.09 and 0.99, *P*<0.001, values were logarithmic transformed for analysis). In CHB-IC patients, HBV replicative efficiency was positively correlated with histological activity index of liver inflammation (*r* = 0.308, *P* = 0.009).

**Conclusion:**

Serum HBsAg and HBV DNA levels may reflect the amount of active intrahepatic cccDNA in treatment-naive AHB and CHB-IC patients. Reduced intrahepatic cccDNA and HBV replicative efficiency may imply effective immune control of HBV infection.

## Introduction

Chronic hepatitis B virus (HBV) infection remains a serious health problem. Around 350–400 million people worldwide and 93 million in China are chronic HBV surface antigen (HBsAg) carriers [1], [2]. Chronic HBV infection is associated with high risk of developing liver cirrhosis and hepatocellular carcinoma [3]. One crucial step in HBV life cycle is the formation of covalently closed circular DNA (cccDNA), which serves as original template for viral replication and plays an important role in the persistence of HBV infection [4], [5], [6]. Quantitation of intrahepatic HBV cccDNA is suggested to be valuable in evaluating anti-HBV therapeutic efficacy and estimating treatment endpoint [7], [8], [9], [10], [11], [12]. In addition, HBV cccDNA level is considered to be a valuable marker for HBV recurrence after liver transplantation and extrahepatic HBV infection [13], [14].

The major obstacle for quantitation of cccDNA in clinic is the requirement for invasive liver biopsy. Therefore, finding serum surrogate maker of intrahepatic cccDNA is clinically meaningful. Recently, correlation between serum HBsAg and HBV DNA levels with intrahepatic cccDNA level has been actively investigated for patients with chronic hepatitis B infection (CHB) [15], [16], [17]. Serum HBsAg and HBV DNA levels were suggested to serve as a surrogate markers of intrahepatic cccDNA for evaluating effectiveness and endpoint of anti-HBV treatment for patients with chronic hepatitis B, particularly for HBeAg-positive patients. However, controversial conclusion existed. For examples, patients on treatment with nucleos(t)ide analogs with greater reductions in levels of cccDNA had greater reductions in HBsAg, but these reductions were not statistically significant [11]; serum HBV DNA, but not HBsAg, reflected the amount of cccDNA and the replicatvive efficiency of HBV in patients with HBeAg-negative hepatitis B [12]. Similarly, inconsistent results were documented on the relationship between intrahepatic cccDNA and serum HBV DNA among HBeAg-positive patients prior to antiviral treatment [7], [16].

The dynamic processes of intrahepatic and serum HBV replication markers and histopathological damage are suggested to be complex in CHB patients [18], [19]. The production of HBsAg by the cccDNA can be independent of the replication of the virus [16], and study results could be influenced by disease stages, antiviral treatments, sample resource and size, and cccDNA quantitative assays. There is still a paucity of data presenting the correlation of intrahepatic cccDNA with serum viral markers in patients with acute hepatitis B (AHB) and treatment-naive CHB patients at different natural phases. This study aimed to investigate the relationship between intrahepatic cccDNA and serum viral markers including HBsAg and HBV DNA throughout natural phases of acute and chronic HBV infection.

## Materials and Methods

### Study Subjects

The study population consisted of 19 AHB and 101 CHB Chinese patients who were admitted to Beijing 302 Hospital from January 2008 to December 2012. CHB patients comprised of 10 in immune-tolerant (IT) phase, 59 in immune-clearance (IC) phase, 8 in low-replicative (LR) phase, and 24 in HBeAg-negative (ENH) phase. The diagnosis criteria for acute and chronic hepatitis B were according to the 2000 Xi’an Viral Hepatitis Management Scheme issued by the Chinese Society of Infectious Diseases and Parasitology and the Chinese Society of Hepatology, of the Chinese Medical Association [20], and had been described in details in our previous studies [21], [22]. The definitions of phases of persistent HBV-infection were shown in Table 1 according to previous definitions [23]. The clinical backgrounds of patients were summarized in Table 2. In the 32 HBeAg-negative patients, 27 harbored G1896A and (or) A1762T+G1764A mutations (19 with G1896A, 18 with A1762T+G1764A, and 10 with the both). HBeAb was positive for 29 of the 32 HBeAg-negative patients. Patients co-infected with hepatitis A, C, D, E, and F virus, or patients with autoimmune or metabolic liver disease were excluded. The study was approved by ethics committee of Beijing 302 Hospital and the written informed consents were obtained from all patients.

**Table 1:** Definitions of phases of persistent HBV infection.

Phase	HBeAg status	HBV DNA (lU/ml)	ALT* (U/L)
immune tolerance (IT)	(+)	>107	<ULN
immune clearance (IC)	(+)	>2000	>2× ULN
low-replicative (LR)	(−)	<2000	<ULN
HBeAg(-) hepatitis (ENH)	(−)	>2000	>2× ULN

*In IT and LR groups patients with ALT activity of <1.5 ULN were accepted if no inflammation in liver biopsy was observed. In IC and ENH groups patients with ALT activity of >ULN were accepted if significant inflammation.

**Table 2:** Clinical details of the studied patients.

	AHB (n = 19)	CHB-IT (n = 10)	CHB-IC (n = 59)	CHB-LR (n = 8)	CHB-ENG (n = 24)	*P* value
Gender (M/F)	15/4	7/3	49/10	4/4	17/7	0.257
Age (years)	32 (24–41)	18 (10–34)	30 (14–43)	30 (15–42)	35 (23–41)	0.332
TBil (µmol/L)	122 (33.9–171.5)	8.4 (5.7–13.1)	12.2 (8.6–24.6)	10.7 (7.8–12.8)	15.0 (8.8–20.4)	<0.001
Albumin (g/L)	39 (36–41)	40 (38–41)	40 (37–43)	39 (38–42.3)	41 (38–46)	0.330
Albumin/globulin	1.4 (1.2–1.8)	1.7 (1.4–1.9)	1.6 (1.3–1.9)	1.5 (1.4–1.8)	1.6 (1.3–1.9)	0.871
Cholinesterase (IU/L)	5661(4270–7225)	7662 (6091–8634)	5899 (4827–7364)	7920 (5788–9544)	6332 (5127–7851)	0.210
ALT (IU/L)	1571(1014–2142)	33 (17–39)	132 (62–398)	20 (17–32)	138 (63–346)	<0.001
HBV DNA (log_10_ IU/mL)	3.88 (2.87–4.80)	7.95 (7.35–8.32)	6.43 (4.86–7.84)	3.08 (2.44–3.14)	6.1 (5.21–8.01)	<0.001
HBeAg	Positive	Positive	Positive	Negative	Negative	
HBV genotype (B/C)	7/12	2/8	11/48	1/7	4/20	

### Sample Collection, Quantitation of Serum HBsAg and HBV DNA

Liver biopsy was obtained from all patients. Sera were collected on the same day of liver biopsy for biochemical, virological and serological analyses. The titer of serum HBsAg was quantitated by a chemiluminescence assay using the Architect i2000SR platform and Abbott Architect HBsAg reagents (Abbott Laboratories, Chicago, IL), according to the manufacture’s instruction. The lower limit of detection was 0.05 IU/mL. If the initial test value was higher than the upper limit of detection (250 IU/mL), the samples were diluted (1∶500) and reassessed. Serum HBV DNA was quantitated by Taqman real-time polymerase chain reaction using commercially available HBV-DNA real-time quantitative PCR kit (Fosun Pharmaceutical Co., Ltd., Shanghai, China) with a lower detection limit of 100 IU/mL, or COBAS AmpliPrep/COBAS TaqMan (Roche Diagnostics, Mannheim, Germany) with a lower detection limit of 12 IU/mL if needed.

### HBV Genotype Classification

HBV genotype assignment as based on phylogenetic analysis of the 1,225-bp-long S/Pol gene sequence (nucleotides 54 to 1278) as we described previously [24]. Phylogenetic and molecular evolutionary analyses were performed in MEGA version 4. Phylogenetic trees were constructed using neighbor-joining (NJ) analysis with bootstrap test confirmation performed on 1,000 resampling standard reference sequences acquired from the online Hepatitis Virus Database (http://www.ncbi.nlm.nih.gov/projects/genotyping/formpage.cgi).

### Histological Examination of Liver Tissues

Histological sections of liver biopsy specimens were stained with hematoxylin-eosin. Histologic grading of necroinflammation and staging of fibrosis were performed using Knodell histological activity index (HAI) (range 0 to 18) and the Ishak fibrosis score (range 0 to 6) respectively, as previously described [18], [25], [26]. The degree of necroinflammation was classified into score 0–3, 4–8, 9–12, and >score 12 groups. The stage of fibrosis was classified into 4 groups based on the Ishak fibrosis scores.

### Quantitation of Intrahepatic HBV cccDNA

Intrahepatic HBV cccDNA was extracted and quantitated by the assay as we described previously [27]. Briefly, about 30 µm formalin fixed paraffin-embedded (FFPE) liver biopsy tissue was sectioned to 6 µm each for DNA extraction. To prevent contamination, disposable tweezers, brush and interleaver were used and sectioning blades were carefully cleaned with 70% ethanol after every sampling. The DNA was extracted using QIAamp FFPE DNA Mini Kit (QIAGEN, GmbH, Hilden, Germany) according to the instructions of the manufacturer. PSAD (Epicentre, Madison, WI, USA) was used to digest HBV rcDNA, replicative dsDNA and ssDNA. Afterwards, rolling Circle Amplification (RCA) was conducted to selectively amplify circle DNA. Using the RCA products as template, HBV cccDNA was further amplified and quantified with TaqMan real-time PCR mediated by a pair of cccDNA-selective primers and a probe that targets the gap region between the two direct repeat regions (DR1 and DR2) of the viral genome. To quantitate cell numbers, a set of primers and a probe for reference control DNA segment of human beta-actin were used in the real-time PCR process. Cell numbers were calculated based on an estimation of 6.667 pg/hgDNA per cell.

### Statistical Analysis

Data were presented as median and inter-quartile range. Differences between variables was tested by Student’s *t*-test or one-way ANOVA test where it is appropriate. Regression and Pearson’s correlation analysis were applied to compare the data obtained by real-time PCR methods. A *P*-value (2-tailed) of <0.05 was considered statistically significant. All statistical analyses were carried out in Statistical Program for Social Sciences (SPSS 18.0 for Windows; SPSS Inc., Chicago, IL).

## Results

### Intrahepatic HBV cccDNA Levels in Different Groups of Patients

**Figure 1:**
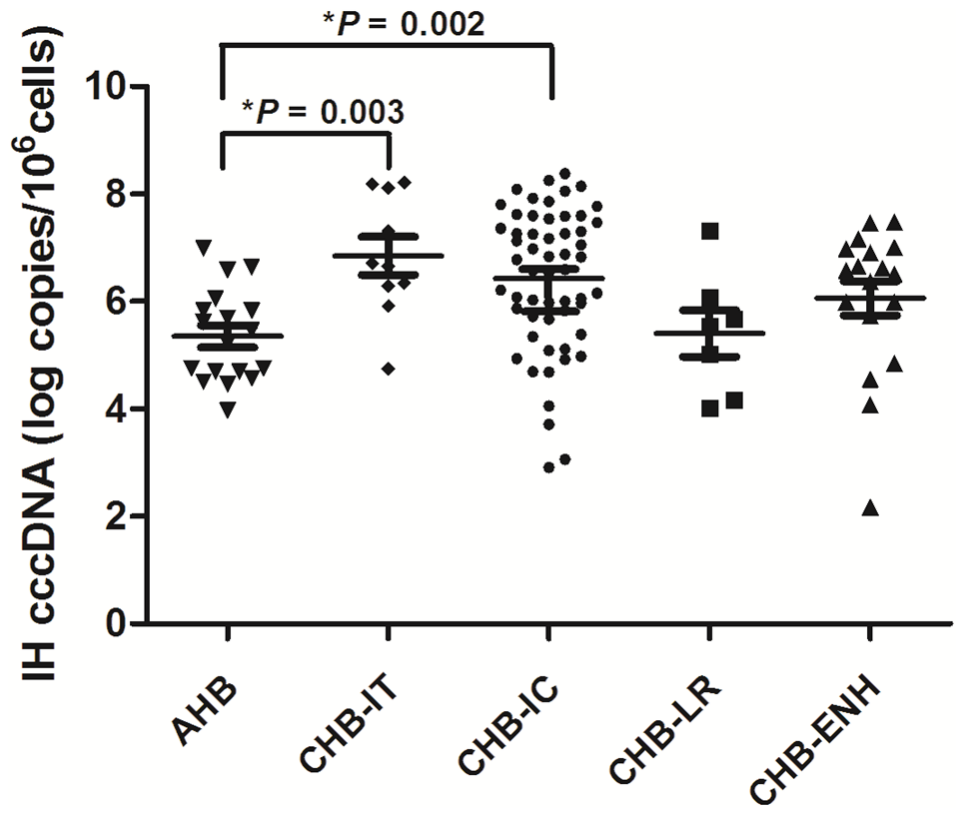
Comparison of intrahepatic (IH) HBV cccDNA levels among patients with different clinical presentations. The horizontal lines illustrate the 25th, 50th, and 75th percentiles of the cccDNA levels. Symbols * and ** represent *P* value less than 0.01 and 0.05, respectively. AHB, acute hepatitis B; CHB, chronic HBV infection; IT, immune-tolerant; IC, immune-clearance; LR, low-replicative; ENH, HBeAg-negative hepatitis.

The median (25th–75th centile) intrahepatic cccDNA levels were 0.17 (0.04–0.68), 4.80 (1.66–136.97), 3.81 (0.23–29.43), 0.22 (0.11–1.02) and 0.97 (0–7.09) copies/cell in patients with AHB, CHB-IT, CHB-IC, CHB-LR, and CHB-ENH, respectively. HBV cccDNA level was significantly lower in patient with AHB than in patients with CHB-IT and CHB-IC patients; CHB-IT and CHB-IC patients had a significantly higher cccDNA level than CHB-LR patients (Figure 1).

### Serum HBsAg Levels in Different Groups of Patients

**Figure 2:**
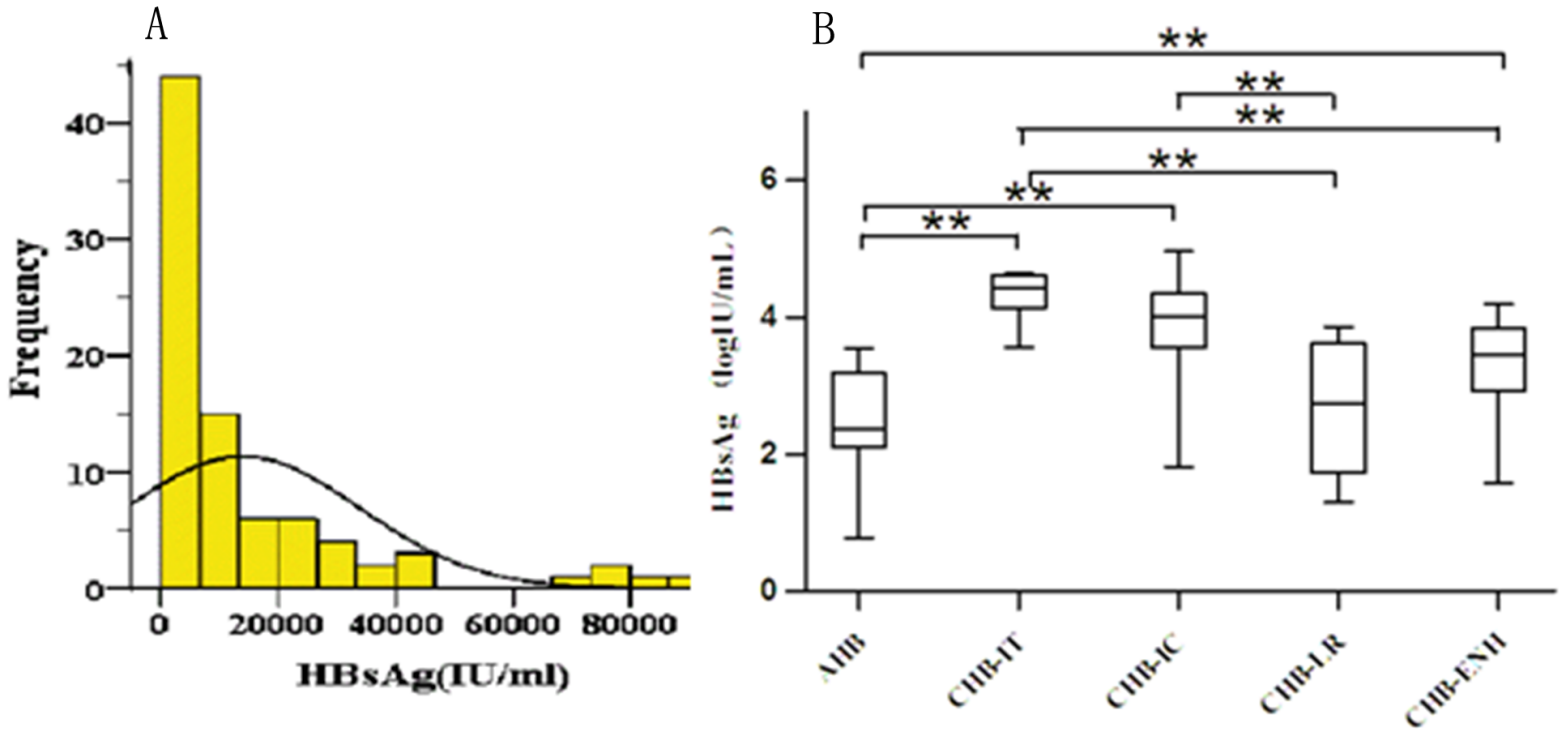
Quantitation of serum HBsAg for 120 HBV-infected patients with different clinical presentations. The distribution of serum HBsAg level across the study population (A). Comparison of serum HBsAg levels among patients with different clinical presentations (B). Symbol ** represent *P* value less than 0.01.

As shown in Figure 2A, the distribution of serum HBsAg level across the study population was skewed (median 3850, range 1.9–91 000 IU/ml in studied population). AHB patients had a significantly lower median serum HBsAg level than CHB patients (186 IU/ml vs. 6,330 IU/ml, *P*<0.001). Specifically, HBsAg level was different between AHB patients and CHB-IT/CHB-IC/CHB-ENH but not CHB-LR patients. The median serum HBsAg levels for CHB patients in different phases were 22 550 IU/ml (range 3 610–43 900 IU/ml) in CHB-IT patients, 8 680 IU/ml (range 2.1–91 000 IU/ml) in CHB-IC, 563 IU/ml (range 2.1–7 210 IU/ml) in CHB-LR patients, and 2 895 IU/ml (range 38–15 400 IU/ml) in ENH patients, respectively. CHB-IT patients had a significantly higher HBsAg level than CHB-LR and CHB-ENH patients. CHB-IC patients had a significantly higher HBsAg level than CHB-LR patients (Figure 2B).

### Correlation of Intrahepatic cccDNA with Serum HBsAg and Serum HBV DNA

**Figure 3:**
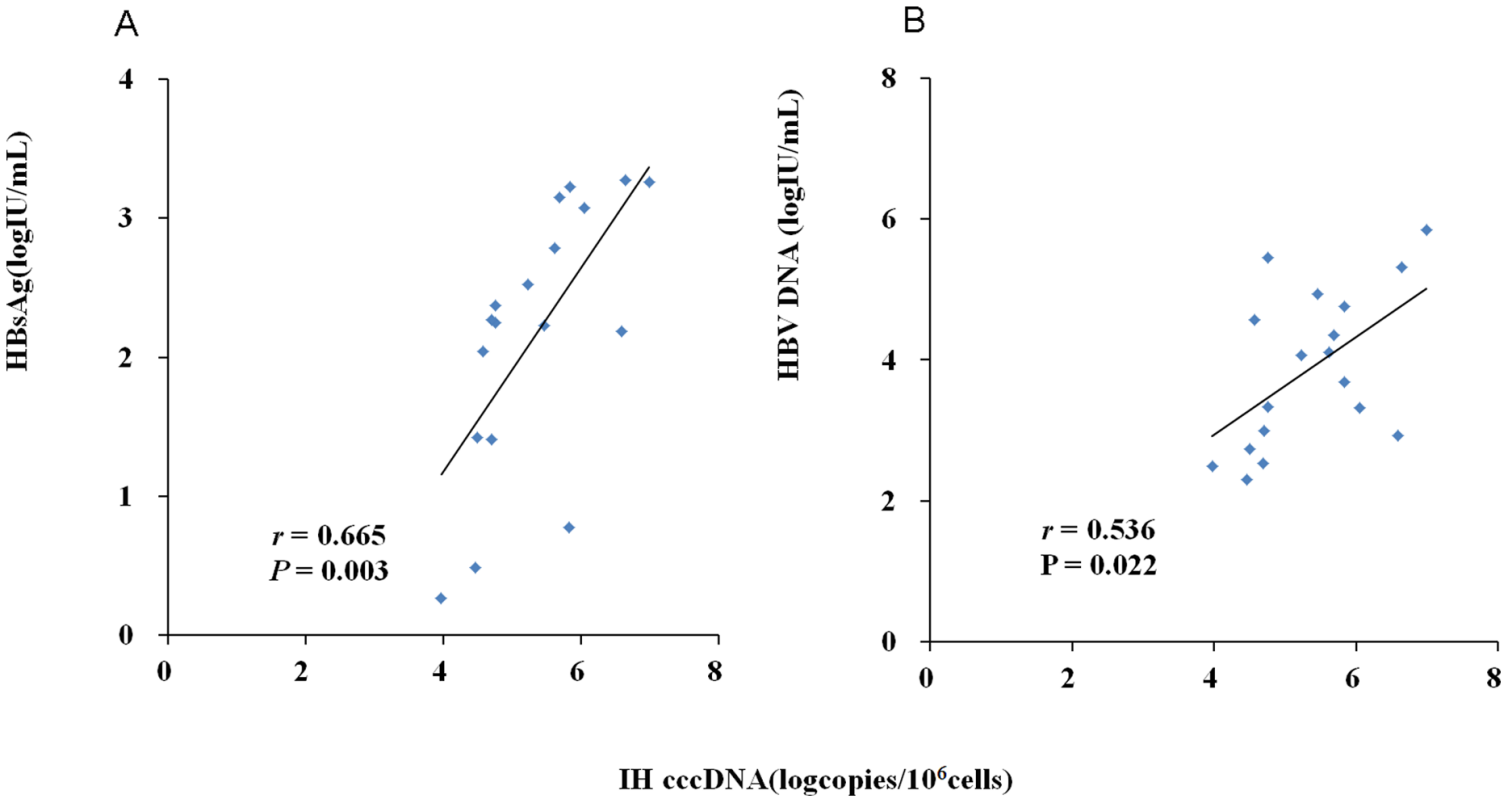
Analysis of correlation between intrahepatic (IH) HBV cccDNA and serum HBV markers for 19 patients with acute hepatitis B. Correlation between intrahepatic HBV cccDNA level and serum HBsAg level (A). Correlation between intrahepatic HBV cccDNA level and serum HBV DNA level (B).

In AHB patients, positive correlation was observed between intrahepatic cccDNA and serum HBV DNA (*r* = 0.536, *P* = 0.022), as well as HBsAg level (*r* = 0.665, *P = *0.003) (Figure 3).

**Figure 4:**
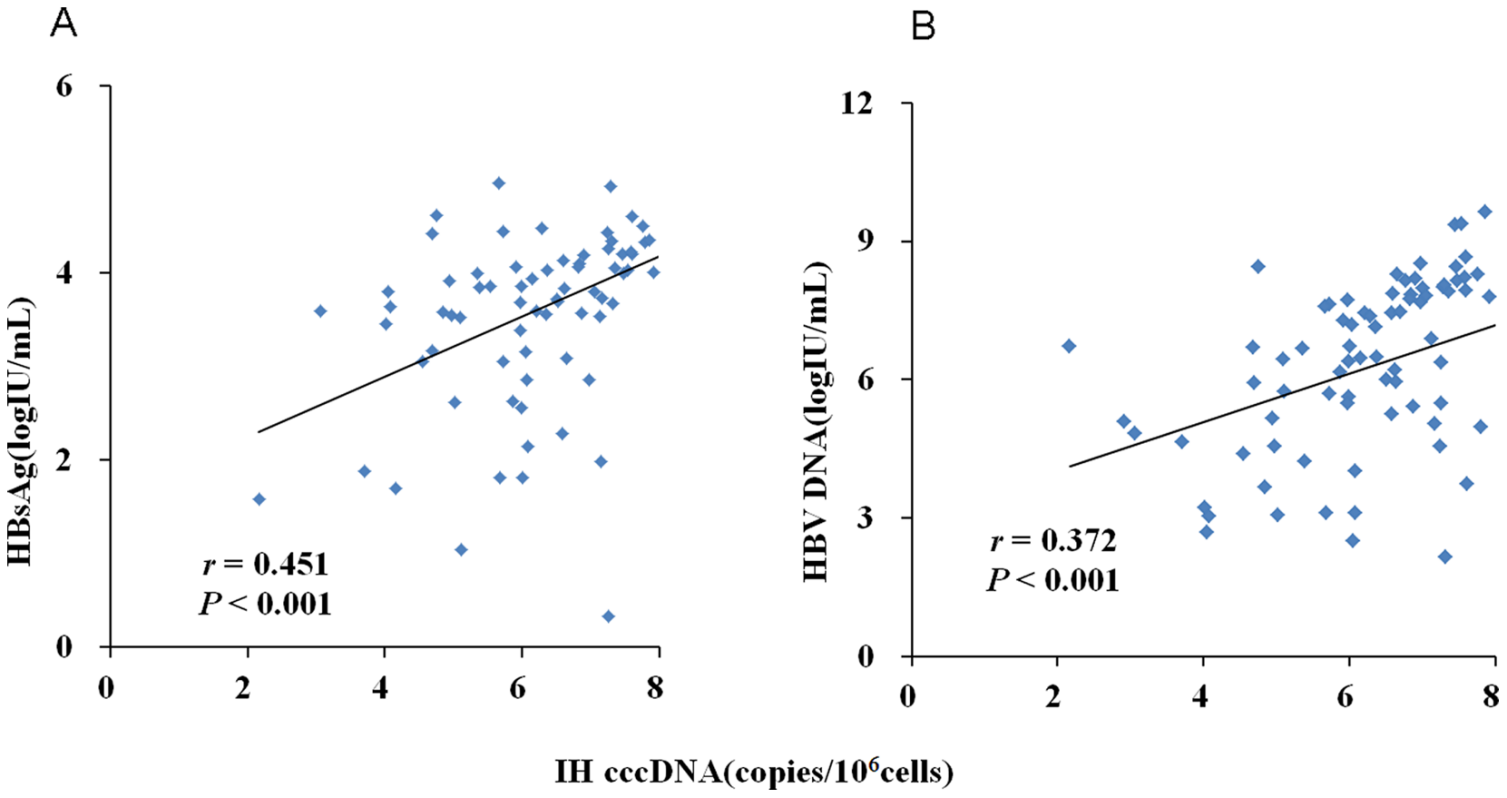
Overall analysis of correlation between intrahepatic (IH) HBV cccDNA and serum HBV markers across 101 patients with chronic HBV infection. Correlation between IH cccDNA level and serum HBsAg level (A). Correlation between IH cccDNA level and serum HBV DNA level (B).

**Figure 5:**
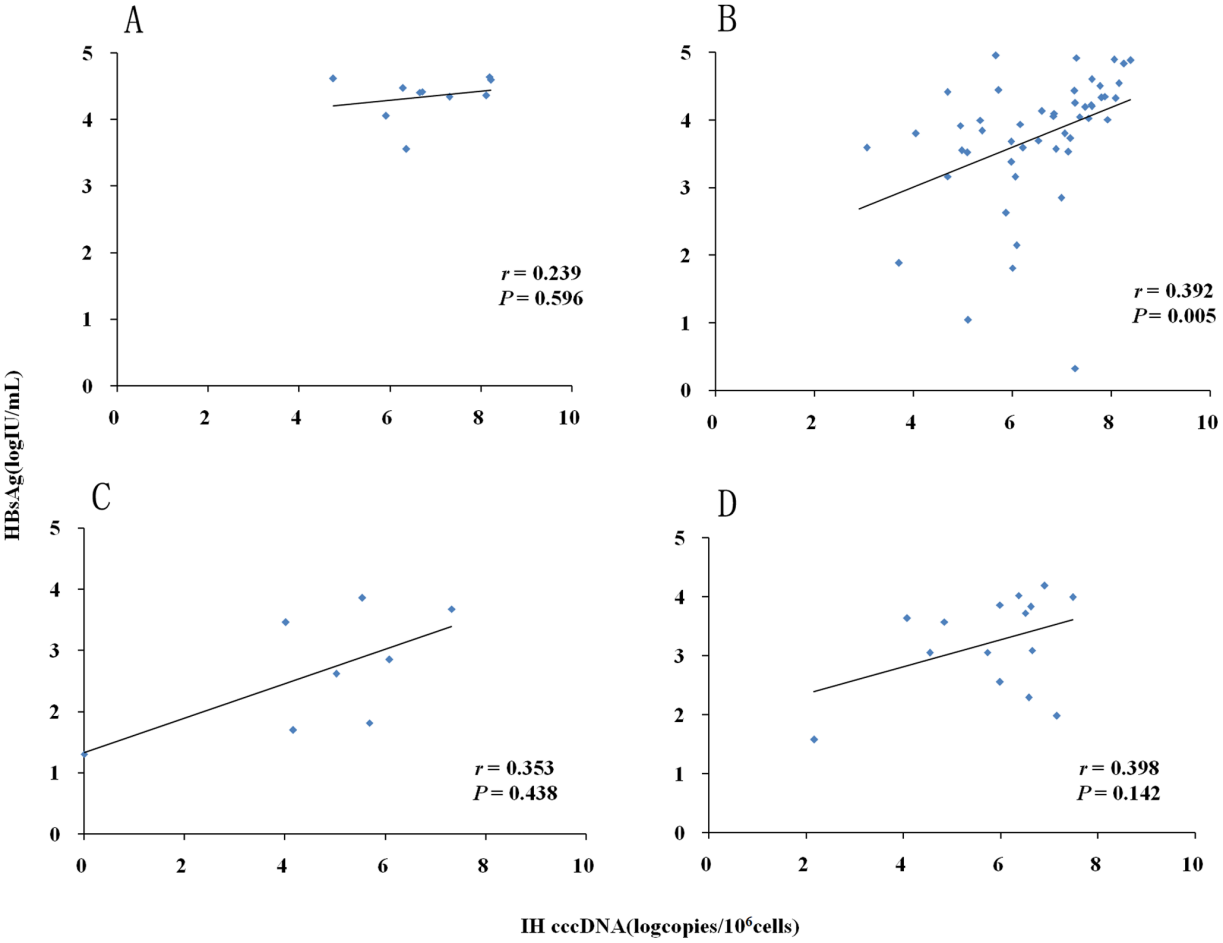
Phase-categorized analysis of correlation between intrahepatic (IH) HBV cccDNA level and serum HBsAg level for patients with chronic HBV infection. Analysis for 10 patients in immune-tolerant phase (A). Analysis for 59 patients in immune-clearance phase (B). Analysis for 8 patients in low-replicative phase (C). Analysis for 24 patients in HBeAg-negative hepatitis phase (D).

**Figure 6:**
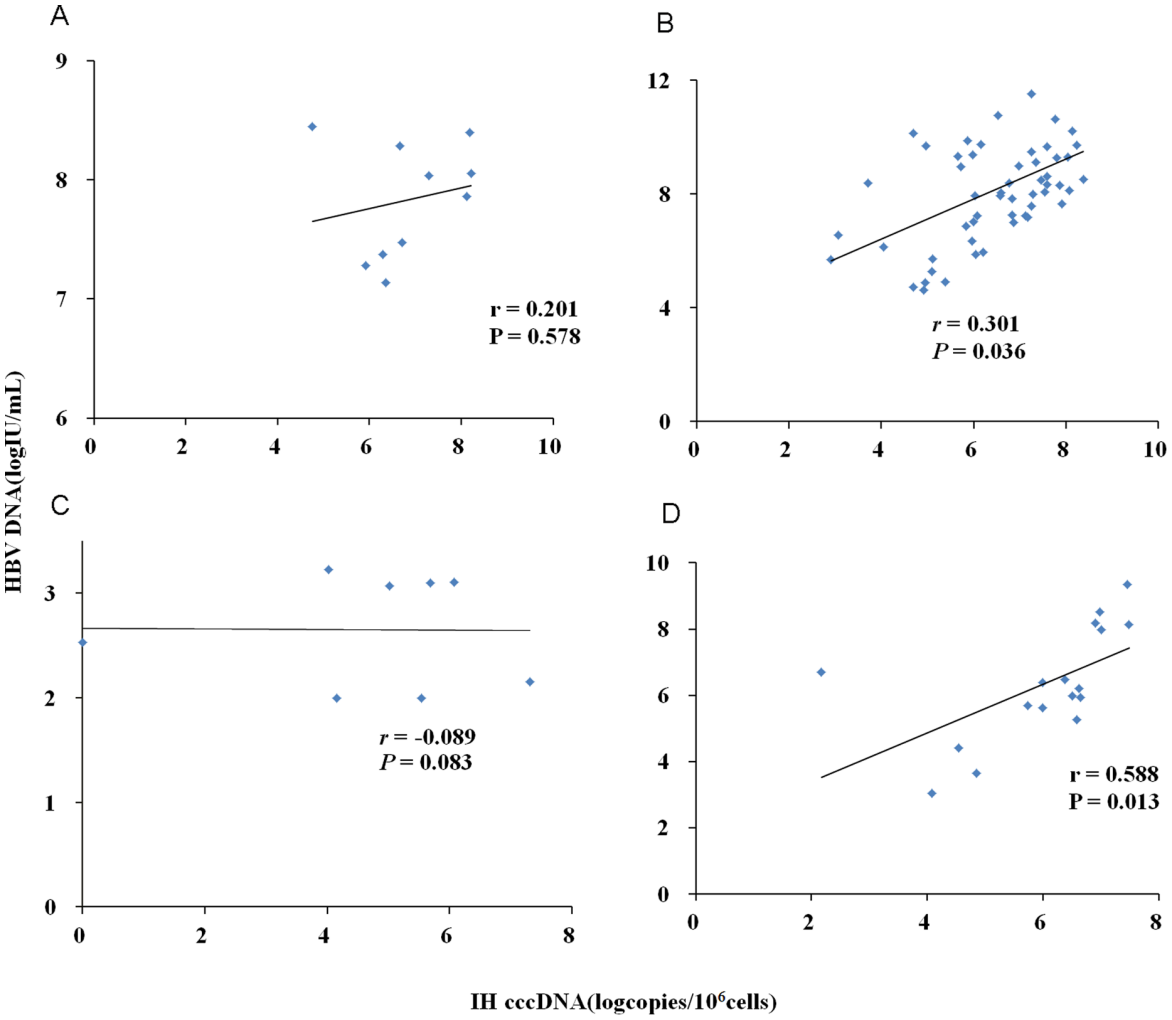
Phase-categorized analysis of correlation between intrahepatic (IH) HBV cccDNA level and serum HBV DNA level for patients with chronic HBV infection in different phases. Analysis for 10 patients in immune-tolerant phase (A). Analysis for 59 patients in immune-clearance phase (B). Analysis for 8 patients in low-replicative phase (C). Analysis for 24 patients in HBeAg-negative hepatitis phase (D).

In the entire cohort of CHB patients, positive correlation was observed between intrahepatic cccDNA level and serum HBsAg level (*r* = 0.451, *P*<0.001), as well as serum HBV DNA level (*r* = 0.372, *P*<0.001) (Figure 4). When analyzing the different phases of patients separately, the significant correlation between intrahepatic cccDNA level and serum HBsAg level was only observed in the IC phase rather than in other three phases (Figure 5); and significant correlation between intrahepatic cccDNA level and serum HBV DNA level was observed in the IC and ENH phases rather than in the IT and LR phases (Figure 6).

### Dynamics of Serum HBsAg and HBV DNA Levels

**Figure 7:**
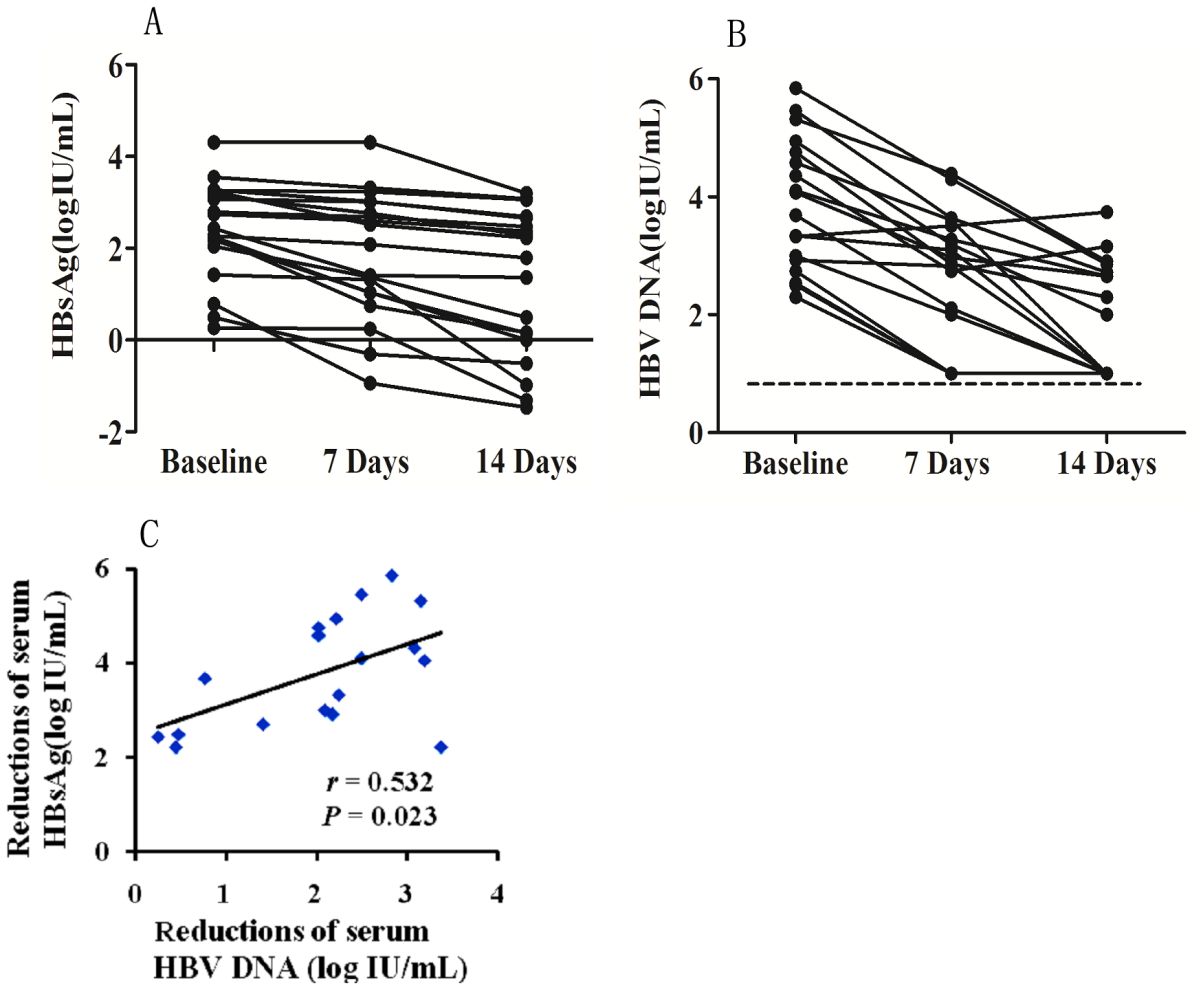
Monitoring of serum HBsAg and HBV DNA levels in patients with acute hepatitis B. Dynamical change of serum HBsAg level (A). Dynamical change of serum HBV DNA level (B). Association of reductions between serum HBsAg level and HBV DNA level 14 days post patient admission (C).

In AHB patients, a progressive reduction of serum HBsAg level was observed, which was closely associated with a rapid decline of serum HBV DNA level (Figure 7). The reduction medians (25th–75th centile) were 0.62 (0.20–2.07) and 2.54 (2.30–2.99) log_10_ IU/mL for serum HBsAg and HBV DNA 14 days post patient admission, respectively. By contrast, in CHB patients. serum HBsAg level changed much slower and its associations with HBV DNA level was weak or missing.

### Comparison of HBV Replicative Efficiency among Different Groups of Patients

HBV replicative efficiency, defined as the ratio of serum HBV DNA to intrahepatic cccDNA (log_10_ serum HBV DNA/log_10_ cccDNA (copies/10^6^ cells) [12], was analyzed. The replicative efficiency medians (25th–75th centile) were 0.70 (0.55–0.81) in AHB patients, 1.12 (1.01–1.24) in CHB-IT patients, 1.09 (0.78–1.20) in CHB-IC patients, 0.53 (0.22–0.66) in CHB-LR patients, and 0.99 (0.91–1.06) CHB-ENH patients. AHB and CHB-LR patients had significantly lower replicative efficiency than CHB-IT, CHB-IC and CHB-ENH patients (*P*<0.001). The values of HBV replicative efficiency were not statistically different among CHB-IT, CHB-IC, and CHB-ENH patients.

In CHB patients, HBV replicative efficiency was positively associated with alanine aminotransferase (ALT) level (*r* = 0.390, *P*<0.001). Further analysis showed that replicative efficiency was correlated with ALT level only in CHB-IC patients (*r* = 0.515, *P*<0.001), but not in CHB-IT patients (*r* = 0.333, *P* = 0.347), CHB-LR patients (*r* = 0.653, *P* = 0.180), or CHB-ENH patients (*r* = 0.221, *P* = 0.393).

**Figure 8:**
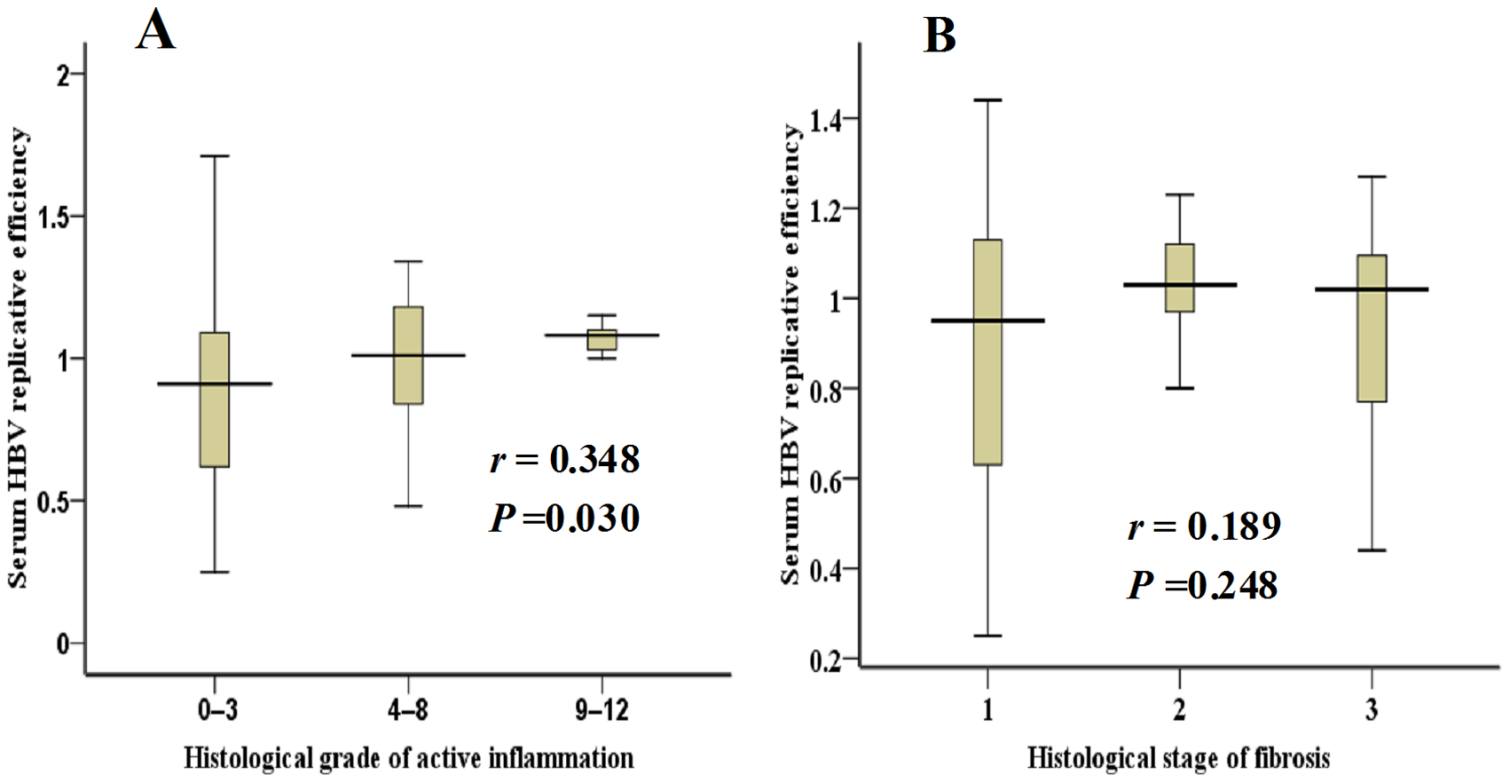
Relationship of HBV replicative efficiency with histological activitiesin CHB-IC patients. Correlation between HBV replicative efficiency and histological grade of active inflammation (A). Correlation between HBV replicative efficiency and histological stage of fibrosis(B).

In CHB-IC patients, HBV replicative efficiency had a correlation with the histological grade of active inflammation (*r* = 0.348, *P* = 0.030). The replicative efficiency medians (25th–75th centile) were 0.97 (0.64–1.14), 1.07 (0.91–1.23), and 1.13 (1.05–1.20) in score 0–3, 4–8 and 9–12 groups, exhibiting a stepwise elevation with the increase of the inflammation grade. By contrast, HBV replicative efficiency was not correlated with stage of fibrosis (r = 0.189, P = 0.248) (Figure 8). In the other 3 phases of CHB patients, no significant correlation was observed between HBV replicative efficiency and the liver histological activities (data not shown).

## Discussion

This study for the first time revealed the comprehensive relationship of intrahepatic cccDNA with serum HBsAg and HBV DNA throughout the natural phases of both acute and chronic HBV infections. Previous studies have not described intrahepatic cccDNA with serum markers in AHB patients, nor CHB patients in the LR phase, owing to the particular difficulty in obtaining liver biopsy from these patients in clinical practice. The current study made a great effort to fill up the knowledge gap.

Intrahepatic cccDNA level was significantly lower in AHB patients than that in CHB patients in this study. Dynamical observation showed that both serum HBsAg and HBV DNA levels progressively declined in a short period of time with a good correlation to each other. These evidence suggested that active anti-HBV immune response in AHB patients could effectively eliminate intrahepatic cccDNA and the effect was reflected well in peripheral. Thus, serum HBsAg and HBV DNA levels could serve as surrogate markers for intrahepatic cccDNA level in AHB patients.

In this study, HBV replicative efficiency was significantly lower in AHB patients than in CHB patients in overall, but CHB-LR patients had a low HBV replicative efficiency value similarly as in AHB patients. Consistently, patients with inactive HBeAg-negative chronic hepatitis B was observed to have a lower cccDNA level and a lower viral replicative efficiency than patients with active HBeAg-negative chronic hepatitis B [12]. This suggested that rate of active cccDNA loss was faster than the rate of total cccDNA loss when HBV activity is well checked by host immunity, such that reduced replicative efficiency may imply effective immune control of HBV infection.

Discrepancy in the relationship between intrahepatic HBV cccDNA level and histological activities has been described from previous studies [12], [18], [28], [29]. In this study, intrahepatic cccDNA level was not correlated with histological activities of liver inflammation or fibrosis. Our results are consistent with that by Lin et al [12] and by Guner et al [18]. The reason for the lack of correlation may be that histopathological damage was developed over time or that damage was mainly caused by differences in immune response [18]. Interestingly, we found that HBV replicative efficiency (ratio of log_10_ serum HBV DNA to log_10_ intrahepatic cccDNA) was correlated with the histological index of inflammation activity and ALT level in CHB-IC patients, suggesting that HBV replicative efficiency was a valuable indicator associated with liver damage in the IC phase.

One limitation of the study is the relatively small sample size for CHB-LR and CHB-IT groups. In addition, HBV cccDNA activity could be influenced by epigenetic regulation, including DNA methylation, histoneacetylation, and microRNAs [30], [31]. Whether these factors influence the association between intrahepatic cccDNA and serum viral markers needs further clarification.

In summary, the major findings of this study are that (i) Intrahepatic cccDNA levels were significantly lower in patients with AHB and CHB-LR than patients with CHB-IT, CHB-IC, and CHB-ENH. (ii) intrahepatic cccDNA level is positively correlated with serum HBsAg level in untreated AHB and CHB-IC patients, and with serum HBV DNA level in the AHB, CHB-IC, and CHB-ENH patients. (iii) Reduced HBV replicative efficiency may imply effective immune control of HBV infection. (iv) In CHB-IC patients, HBV replicative efficiency is positively correlated with inflammation in the liver. These findings provided new insights on the clinical implications of intrahepatic cccDNA and the serum viral markers in different stages of HBV infection.
