# Correction: Analysis of Hepatitis B Virus Intrahepatic Covalently Closed Circular DNA and Serum Viral Markers in Treatment-Naive Patients with Acute and Chronic HBV Infection

**DOI:** 10.1371/journal.pone.0096382

**Published:** 2014-04-22

**Authors:** The PLOS ONE Staff

The tenth author’s name is spelled incorrectly. The correct name is: Weiming Yao.

The sixth sentence of the “Study Subjects” sub-section of the Materials and Methods is incorrect. The correct sentence should read: In the 32 HBeAg-negative patients, 27 harbored G1896A and (or) A1762T+G1764A mutations (9 with single G1896A, 8 with single A1762T+G1764A, and 10 with the both).

The fifth column header in the first line of Table 2 is incorrect. The correct header is: CHB-ENH (n = 24)

The first sentence in the Results section is incorrect. The correct sentence should read: The median (25th–75th centile) intrahepatic cccDNA levels were 0.18 (0.04–0.68), 4.80 (1.66–136.97), 3.81 (0.23–29.43), 0.22 (0.11–1.02) and 0.97 (0–7.09) copies/cell in patients with AHB, CHB-IT, CHB-IC, CHB-LR, and CHB-ENH, respectively.

There are five instances in which log10 is incorrectly displayed. The correct format is: log**_10._**
